# High correlation between salivary cortisol awakening response and the psychometric profiles of healthy children

**DOI:** 10.1186/1751-0759-8-9

**Published:** 2014-03-14

**Authors:** Ikuhiko Shibuya, Shinichiro Nagamitsu, Hisayoshi Okamura, Shuichi Ozono, Hiromi Chiba, Takashi Ohya, Yushiro Yamashita, Toyojiro Matsuishi

**Affiliations:** 1 Department of Pediatrics and Child Health, Kurume University School of Medicine, 67 Asahi-machi, Kurume, Fukuoka 830-0011, Japan; 2 Cognitive and Molecular Research Institute of Brain Diseases, Kurume University School of Medicine, Kurume, Japan; 3 Department of Psychiatry, Kurume University School of Medicine, Kurume, Japan

**Keywords:** Cortisol awakening response, Hypothalamus-pituitary-adrenal axis, Saliva, Children

## Abstract

**Background:**

Cortisol awakening response (CAR) as an indicator of psychological stress and related physical and psychiatric diseases has attracted growing attention from researchers. Although CAR changes have been investigated extensively in children with behavioral and psychiatric disorders, the association between CAR and conventional psychometric scales for healthy children has not been reported. The aim of this study was to investigate the association between salivary CAR and subscales of Profiles of Mood States (POMS), a self-assessment questionnaire widely used to evaluate the temporal emotional states of healthy children.

**Findings:**

This study included 18 healthy girls aged 13–16 years. Saliva was collected immediately on awakening, 30 min and 60 min after waking, and then at 2-hour intervals from 9 am to 5 pm. The current mood state, including depression, anxiety, fatigue, and other psychometric profiles were assessed using POMS. The magnitude of salivary CAR and the area under the concentration-time curve (AUC) for diurnal salivary cortisol were compared with the profiles. There were significant positive correlations between the magnitude of CAR and the POMS subscales for "Depression-Dejection", "Tension-Anxiety", "Fatigue", and "Confusion". No correlation was found between the AUC salivary cortisol level and the psychometric profiles.

**Conclusions:**

Salivary CAR was associated with various mood states of healthy female children but diurnal salivary cortisol AUC was not. Salivary CAR may be a biomarker of the physical and mental condition of healthy female children.

## Findings

The hypothalamic-pituitary-adrenal (HPA) axis is a major homeostatic system that maintains an organism’s equilibrium within its environment [1]. The HPA axis is the primary mammalian system of stress response, and the endpoint of HPA-axis activation is the release of the glucocorticoid cortisol. The principal role of cortisol during the response to stress is to restrain the effectors of the stress response [2]. Cortisol secretion is governed by a diurnal rhythm; levels are at their highest in the morning and gradually decrease during the night. In addition to the circadian variation, there is an acute increase of cortisol secretion after awakening [3,4]. This cortisol awakening response (CAR) may be a more appropriate measure than cortisol concentration for assessing HPA activation in relation to psychosocial factors.

Several studies have provided evidence of an association between HPA dysregulation and psychiatric symptoms. Depressed adults show increased total cortisol secretion, and a flattened diurnal rhythm of cortisol production is characteristic of HPA-axis hyperactivity [5]. Young people at familial risk of depression but with no personal history of mood disorders have higher cortisol secretion compared with controls with no familial history of depression, indicating that elevated cortisol secretion may serve as a vulnerability marker of major depression [6]. Increased HPA-axis activity has been reported in children with anorexia nervosa, a condition in which cortisol concentration is positively associated with the severity of illness, and relative change in cortisol predicts disease prognosis [7].

The prevalence of childhood stress and the psychosomatic and emotional symptoms of school-aged children have received much attention recently [8]. Parent-reported data on childhood adversities and psychosomatic and emotional symptoms collected for 4,066 children from eight European countries showed that 45.7% of the children experienced at least one those symptoms [9]. Similarly, a cross-sectional survey in China showed over one-third of the children (n = 2,191) reported psychosomatic symptoms at least once per week (37% headache and 36% abdominal pain) [10]. The high prevalence of psychosomatic symptoms in population-based samples might indicate that most children have a predisposition to develop emotion-related psychosomatic and behavioral disorders in stressful environments. An association between HPA function and the psychiatric and behavioral symptoms of children has been reported. Raine reported an association between low basal HPA activity and high levels of disruptive behavior in children, suggesting that individuals with low arousal levels may seek stimulation [11]. Another report revealed that higher HPA activity was associated with persistent anxiety problems in children [12]. Prolonged elevations in cortisol levels may damage hippocampal neurons and affect the glucocorticoid feedback inhibition of corticotropin releasing hormone (CRH) secretion resulting in higher CRH and cortisol concentrations [13].

Although CAR changes and their effects on HPA activities have been reported in children with psychosomatic symptoms, the association of HPA activities and CAR with the physical and mental conditions of healthy children is not well studied. To investigate this association, we compared salivary cortisol concentration after awakening and during the diurnal period with the Profiles of Mood States (POMS) subscales.

The subjects included in this study were 18 females with no history of psychiatric disease, organic disease, psychiatric treatment, or endocrine disease. Participants were enrolled from one public junior high school and participated in this study voluntarily. Only female subjects were enrolled to exclude the sex differences in the gonadotropic hormone effects on cortisol secretion. Another reason was to try to ensure better compliance with the timing of salivary sampling after awakening. Their average age and body mass index were 14.3 ± 1.0 years and 19.3 ± 2.2 kg/m^2^ (mean ± standard deviation (SD)), respectively. The Ethics Committee of Kurume University School of Medicine approved the study protocol, and written informed consent was obtained from each subject.

Salivary samples were collected eight times on a weekend day; at awakening, at 30 min and 60 min after awakening, and at 2-hour intervals from 9 am to 5 pm (0900 h, 1100 h, 1300 h, 1500 h, and 1700 h). All participants arose by 9 am. The samples were collected at home. To avoid the influence of food intake on the cortisol level, the participants were asked to eat breakfast after the awakening cortisol sample was collected. Subjects were requested to stay at home during weekends for saliva sampling. Saliva was not collected during menstruation. Subjects were instructed not to collect saliva within 30 minutes after eating, drinking, walking, or teeth brushing. Salivary sampling is a well-established technique for cortisol measurement for adults and children. Sampling of saliva was conducted using Salisoft^®^ Assist Co. Ltd., Tokyo). A cotton swab was chewed for 3 minutes, and then inserted into a double-chamber plastic test tube. Saliva samples were centrifuged at 4°C and stored at -80°C until required for assay. Salivary cortisol was measured by an enzyme immunoassay (high sensitivity salivary cortisol ELISA kit; Salimetrics LLC, USA). The limit of detection of this assay in our laboratory was 0.19 nmol/liter, and the intra- and inter-assay coefficients of variation were 5.43% and 6.41%, respectively [14].

At the end of the cortisol sampling day, the participants rated their mood using the short form of the POMS to evaluate whether mood disturbances were associated with the cortisol level. The POMS assessment is an excellent measure of the fluctuating affective mood state [15]. The short form of POMS consists of 30 items describing six moods: "Tension-Anxiety", "Depression-Dejection", "Anger-Hostility", "Vigor", "Fatigue’, and "Confusion". Each original POMS score was converted to a T-score [16].

For each subject, CAR was calculated by subtracting cortisol concentration at awakening from cortisol concentration at 30 min after awakening. Each subject’s diurnal cortisol concentration from awakening to 5 pm was expressed as the area under the concentration-time curve (AUC) [17]. Pearson’s correlation was used to analyze the correlation. *p* values less than 0.05 were considered statistically significant.

**Figure 1:**
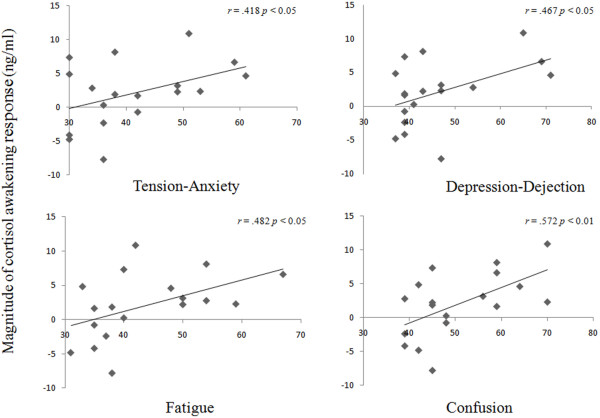
**Correlations between the magnitude of CAR and the subscale scores of POMS.** High POMS subscale scores demonstrated statistically significant associations with elevated CAR. CAR: cortisol awakening response, POMS: profile of mood states.

The average CAR concentration was 2.03 ± 4.67 ng/ml (mean ± SD), (range -7.78 – 10.85 ng/ml), similar to the level of a previous report [18]. The average scores for each of the subscales of POMS were 41.3 ± 10.1 (Tension-Anxiety), 46.4 ± 11.1 (Depression-Dejection), 47.4 ± 6.2 (Anger-Hostility), 46.1 ± 6.7 (Vigor), 43.7 ± 10.1 (Fatigue), and 50.8 ± 10.5 (Confusion). The scores were within the normal ranges for the POMS subscales. Significant positive correlations were found between the magnitude of CAR and the POMS subscales "Tension-Anxiety" (*r* = 0.418; *p* < 0.05), "Depression-Dejection" (*r* = 0.467; *p* < 0.05), "Fatigue" (*r* = 0.482; *p* < 0.05) and "Confusion" (*r* = 0.572; *p* < 0.01) (Figure 1). No correlation was found between the AUC salivary cortisol level and the psychometric profiles.

The present study revealed that salivary CAR, but not the measurement of AUC, was significantly correlated with four out of six subscales of POMS, a widely used self-assessment questionnaire. To our knowledge, this is the first report to show an association between salivary CAR and psychometric scales in a cohort of healthy children.

Higher salivary CAR is associated with the depression and anxiety disorders of children [12,18-20]; however the association between the CAR level and depressive and anxiety symptoms of the general child population is not linear. Greaves-Lord *et al.*[12] reported no association between current anxiety problems and CAR in the general child population (n = 1,768), and Bosch *et al.*[19] also reported no association between overall depressive symptoms and CAR in a group of 2,049 preadolescents (mean age 11.1 years). On the other hand, Dietrich *et al.*[18] reported a very weak association between higher cortisol levels and depressive problems in a general-population sample of children (n = 1,604). One possible explanation for these negative or weak associations in population-based samples is the methodological difficulty of maintaining consistent sampling conditions in these large populations, particularly the lack of adjustment for time of awakening. Another explanation is that the association might depend on the psychometric instrument used to identify the subject’s mood states. For instance, the study by Bosch *et al.*[19] used the 13 items of the Affective Problem Scale of Youth Self-Report to assess children’s depressive symptoms. These were divided into a somatic subscale (lack of appetite, trouble sleeping, and others) and a cognitive-affective subscale (loss of pleasure, sadness, and others). The results showed no association between CAR and overall depressive symptoms, but weak associations between CAR and somatic (positive association) and cognitive-affective (negative association) depressive symptoms. The POMS subscales "Depression-Dejection", "Tension-Anxiety", "Fatigue", and "Confusion" are composed of either somatic or cognitive-affective questions (not a mixture of both); therefore, the CAR and psychometric subscales of POMS were correlated in the current study.

Our results indicate that HPA-axis activation has a strong association with the somatic and affective symptoms in healthy children. It also showed no association between diurnal cortisol concentration and the psychometric subscales of POMS. CAR is controlled somewhat independently to cortisol output during the remainder of the day, and associations between the CAR and diurnal cortisol concentration seem to be low [21]. Excessive or chronic stressful events might cause decreased diurnal cortisol secretion due to hypo-activation of the HPA [22]; however, CAR may reflect recent or upcoming daily general stressful events [23,24].

In conclusion, the measurement of salivary CAR would be a useful biomarker for assessing the psychosocial stress of healthy girls. Future studies are expected to identify the association in healthy boys. This biopsychological evidence contributes to the validation of psychometric assessment.

## Abbreviations

AUC: Area under the concentration-time curve; CAR: Cortisol awakening response; CRH: Corticotropin releasing hormone; ELISA: Enzyme-linked immunosorbent assay; HPA: Hypothalamic-pituitary-adrenal; POMS: Profiles of mood states.

## Competing interests

The authors declare that they have no competing interests.

## Authors’ contributions

IS and SN participated in the design of this study and compiled the manuscript. HO carried out measurement of salivary cortisol. SO and HC collected salivary samples. YY and TM supervised the writing of the manuscript. All authors read and approved the final version of this manuscript.

## References

[B1] O’Connor TM O’Halloran DJ Shanahan F The stress response and the hypothalamic-pituitary-adrenal axis: from molecule to melancholia QJM 2000 8 323 333 10.1093/qjmed/93.6.323 10873181

[B2] Jessop DS Turner-Cobb JM Measurement and meaning of salivary cortisol: a focus on health and disease in children Stress 2008 8 1 14 10.1080/10253890701365527 17853059

[B3] Pruessner JC Wolf OT Hellhammer DH Buske-Kirschbaum A von Auer K Jobst S Kaspers F Kirschbaum C Free cortisol levels after awakening: a reliable biological marker for the assessment of adrenocortical activity Life Sci 1997 8 2539 2549 10.1016/S0024-3205(97)01008-4 9416776

[B4] Steptoe A Fink G Cortisol Awakening Response Encyclopedia of Stress, Volume volume 1 2007 2 Oxford: Academic 649 653

[B5] Burke HM Davis MC Otte C Mohr DC Depression and cortisol responses to psychological stress: a meta-analysis Psychoneuroendocrinology 2005 8 846 856 10.1016/j.psyneuen.2005.02.010 15961250

[B6] Mannie ZN Harmer CJ Cowen PJ Increased waking salivary cortisol levels in young people at familial risk of depression Am J Psychiatry 2007 8 617 621 10.1176/appi.ajp.164.4.617 17403975

[B7] Shibuya I Nagamitsu S Okamura H Komatsu H Ozono S Yamashita Y Matsuishi T Changes in salivary cortisol levels as a prognostic predictor in children with anorexia nervosa Int J Psychophysiol 2011 8 196 201 10.1016/j.ijpsycho.2011.08.008 21906636

[B8] Tanaka H Terashima S Borres MP Thulesius O Psychosomatic problems and countermeasures in Japanese children and adolescents Biopsychosoc Med 2012 8 6 10.1186/1751-0759-6-6 22433184 PMC3362750

[B9] Vanaelst B De Vriendt T Ahrens W Bammann K Hadjigeorgiou C Konstabel K Lissner L Michels N Molnar D Moreno LA Reisch L Siani A Sioen I De Henauw S Prevalence of psychosomatic and emotional symptoms in European school-aged children and its relationship with childhood adversities: results from the IDEFICS study Eur Child Adolesc Psychiatry 2012 8 253 265 10.1007/s00787-012-0258-9 22350132

[B10] Hesketh T Zhen Y Lu L Dong ZX Jun YX Xing ZW Stress and psychosomatic symptoms in Chinese school children: cross-sectional survey Arch Dis Child 2010 8 136 140 10.1136/adc.2009.171660 20133328

[B11] Raine A Autonomic nervous system factors underlying disinhibited, antisocial, and violent behavior. Biosocial perspectives and treatment implications Ann N Y Acad Sci 1996 8 46 59 10.1111/j.1749-6632.1996.tb32508.x 8853591

[B12] Greaves-Lord K Ferdinand RF Oldehinkel AJ Sondeijker FE Ormel J Verhulst FC Higher cortisol awakening response in young adolescents with persistent anxiety problems Acta Psychiatr Scand 2007 8 137 144 10.1111/j.1600-0447.2007.01001.x 17650276

[B13] Young EA Akana S Dallman MF Decreased sensitivity to glucocorticoid fast feedback in chronically stressed rats Neuroendocrinology 1990 8 536 542 10.1159/000125388 2162011

[B14] Brydon L Walker C Wawrzyniak A Whitehead D Okamura H Yajima J Tsuda A Steptoe A Synergistic effects of psychological and immune stressors on inflammatory cytokine and sickness responses in humans Brain Behav Immun 2009 8 217 224 10.1016/j.bbi.2008.09.007 18835437 PMC2637301

[B15] Yoshihara K Hiramoto T Sudo N Kubo C Profile of mood states and stress-related biochemical indices in long-term yoga practitioners Biopsychosoc Med 2011 8 6 10.1186/1751-0759-5-6 21635790 PMC3125330

[B16] McNair DM Lorr M Droppleman LF Manual for the Profile of Mood States 1971 San Diego: Educational and Industrial Testing Services

[B17] Fekedulegn DB Andrew ME Burchfiel CM Violanti JM Hartley TA Charles LE Miller DB Area under the curve and other summary indicators of repeated waking cortisol measurements Psychosom Med 2007 8 651 659 10.1097/PSY.0b013e31814c405c 17766693

[B18] Dietrich A Ormel J Buitelaar JK Verhulst FC Hoekstra PJ Hartman CA Cortisol in the morning and dimensions of anxiety, depression, and aggression in children from a general population and clinic-referred cohort: an integrated analysis. The TRAILS study Psychoneuroendocrinology 2013 8 1281 1298 10.1016/j.psyneuen.2012.11.013 23237815

[B19] Pruessner M Hellhammer DH Pruessner JC Lupien SJ Self-reported depressive symptoms and stress levels in healthy young men: associations with the cortisol response to awakening Psychosom Med 2003 8 92 99 10.1097/01.PSY.0000040950.22044.10 12554820

[B20] Bosch NM Riese H Dietrich A Ormel J Verhulst FC Oldehinkel AJ Preadolescents’ somatic and cognitive-affective depressive symptoms are differentially related to cardiac autonomic function and cortisol: the TRAILS study Psychosom Med 2009 8 944 950 10.1097/PSY.0b013e3181bc756b 19834052

[B21] Schmidt-Reinwald A Pruessner JC Hellhammer DH Federenko I Rohleder N Schürmeyer TH Kirschbaum C The cortisol response to awakening in relation to different challenge tests and a 12-hour cortisol rhythm Life Sci 1999 8 1653 1660 10.1016/S0024-3205(99)00103-4 10328525

[B22] Miller GE Chen E Zhou ES If it goes up, must it come down? Chronic stress and the hypothalamic-pituitary-adrenocortical axis in humans Psychol Bull 2007 8 25 45 17201569 10.1037/0033-2909.133.1.25

[B23] Chida Y Steptoe A Cortisol awakening response and psychosocial factors: a systematic review and meta-analysis Biol Psychol 2009 8 265 278 10.1016/j.biopsycho.2008.10.004 19022335

[B24] Fries E Dettenborn L Kirschbaum C The cortisol awakening response (CAR): facts and future directions Int J Psychophysiol 2009 8 67 73 10.1016/j.ijpsycho.2008.03.014 18854200

